# CXCR4/CXCL12/CXCR7 axis is functional in neuroendocrine tumors and signals on mTOR

**DOI:** 10.18632/oncotarget.7738

**Published:** 2016-02-26

**Authors:** Luisa Circelli, Concetta Sciammarella, Elia Guadagno, Salvatore Tafuto, Marialaura del Basso de Caro, Giovanni Botti, Luciano Pezzullo, Massimo Aria, Valeria Ramundo, Fabiana Tatangelo, Nunzia Simona Losito, Caterina Ieranò, Crescenzo D'Alterio, Francesco Izzo, Gennaro Ciliberto, Annamaria Colao, Antongiulio Faggiano, Stefania Scala

**Affiliations:** ^1^ Molecolar Immunology and Immuneregulation, Istituto Nazionale per lo Studio e la Cura dei Tumori - IRCCS Naples “Fondazione G. Pascale”, Naples, Italy; ^2^ Departments of Clinical Medicine and Surgery, “Federico II” University of Naples, Italy; ^3^ Advanced Biomedical Sciences, Division of Pathology, “Federico II” University of Naples, Italy; ^4^ Abdominal Oncology, Istituto Nazionale per lo Studio e la Cura dei Tumori - IRCCS Naples “Fondazione G. Pascale”, Naples, Italy; ^5^ Thyroid and Parathyroid Surgery Unit, Istituto Nazionale per lo Studio e la Cura dei Tumori - IRCCS Naples “Fondazione G. Pascale”, Naples, Italy; ^6^ Economics and Statistics, “Federico II” University of Naples, Naples, Italy; ^7^ Pathology, Istituto Nazionale per lo Studio e la Cura dei Tumori - IRCCS Naples “Fondazione G. Pascale”, Naples, Italy; ^8^ Abdominal Surgery, Istituto Nazionale per lo Studio e la Cura dei Tumori - IRCCS Naples “Fondazione G. Pascale”, Naples, Italy; ^9^ Scientific Directorate, Istituto Nazionale per lo Studio e la Cura dei Tumori - IRCCS Naples “Fondazione G. Pascale”, Naples, Italy

**Keywords:** chemokine, mTOR, NET, clinical outcome, grading

## Abstract

**Objective:**

To evaluate the possible crosstalk between C-X-C chemokine receptor 4 (CXCR4)/C-X-C motif chemokine 12 (CXCL12)/C-X-C chemokine receptor 7 (CXCR7) axis with the mammalian target of rapamycin (mTOR) pathway in neuroendocrine tumors (NETs).

**Methods:**

Sixty-one human NETs were included into the study. CXCR4/CXCL12/CXCR7 axis and mTOR pathway were assessed by qRT-PCR and immunohistochemistry (IHC). The effect of mTOR inhibitor, RAD001, was evaluated on CXCR4 pathway through proliferation and p-Erk and p-AKT induction. Results: CXCR4/CXCL12/CXCR7 axis and p-mTOR were found to be active and correlated with grading, Ki67 index and tumor stage. mTOR pathway activation significantly correlated with poor prognosis. In human NET cells, CXCL12 induced mTOR signalling while AMD3100 (CXCR4-antagonist) impaired it. The mTOR-antagonist, RAD001, impaired the CXCL12-dependent induction of CXCR4 downstream effectors. Combination of AMD3100 and RAD001 potentiate cell growth inhibition.

**Conclusions:**

CXCR4/CXCL12/CXCR7 axis is active in NETs and signals on mTOR. CXCR4 might be considered a prognostic factor in NETs. Combined treatment with AMD3100 and RAD001 may provide clinical benefits in NET patients with drug-resistant.

## INTRODUCTION

Neuroendocrine tumors are relatively rare (incidence about 1-5 cases/100,000/year) and heterogeneous neoplasms with increasing incidence and prevalence [1]. NETs display variegate biological and clinical course in which primary site, stage and grading affect prognosis. According to the last classification of the World Health Organization (WHO) 2010, gastroenteropancreatic NETs (GEP-NETs) are classified into low (G1) intermediate (G2) and high-grade (G3) lesions, based on mitotic count and Ki67 score [2, 3]. High grade lesions (neuroendocrine carcinomas, NEC) present a mitotic count of >20 for 10 high-powered fields (HPF) or a Ki67 proliferation index of >20% [4], and are generally treated with platinum-based chemotherapy regimens. On the contrary, well-differentiated, low- and intermediate-grade NETs (G1, G2) are clinically indolent with a low proliferation index and good prognosis. Approximately 50% of NETs present with metastases at the diagnosis; the conventional treatment is based on biological therapies (somatostatin analogues, α2b-interferon) or chemotherapy. Nevertheless no significant impact on survival has been obtained [5].

Medullary thyroid carcinoma (MTC) is a malignant tumor of the thyroid (5 to 10% of all thyroid malignancies) originating in C-cell, deriving from the neural crest, expressing neuroendocrine markers (chromogranine and synaptofisin). Stage in thyroid cancer is based on TNM Classification and separate stage groupings are recommended for different histotypes [6, 7]. MTC is a well differentiated NET, slowly growing and insensitive to chemotherapy. Somatostatin analogues and interferon only ameliorates symptoms, while the newly identified TK-inhibitors vandetanib and cabozantinib have antiproliferative effect [8-11].

As of today mammalian target of rapamycin (mTOR) inhibitors, such as RAD001, have been approved as second-line therapy in patients with progressive pancreactic NETs [12-14]. mTOR is a serine-threonine kinase regulating different cellular processes, such as metabolism, nutrient sensing, translation and cell growth [15]. Both mTOR molecular complexes, mTORC1 and mTORC2, use mTOR as the catalytic subunit. mTORC1 increases cell growth and proliferation through ribosomal protein S6 kinase 1 (S6K1) and eukaryotic translation initiation factor 4E (eIF4E)-binding protein (4EBP1) [16, 17]. mTORC2 phosphorylates Protein kinase B (Akt), at Thr450 and Ser473 [18, 19]. Evidence suggest that the phosphatidylinositol 3-kinase (PI3K)/Akt/mTOR signaling is involved in NET tumorigenesis and progression, given its critical role in cell proliferation and angiogenesis. Tamburrino et al. showed that the PI3K/Akt/mTOR pathway is crucial to MTC tumorigenesis [20] and in a recent review, Manfredi et al. showed that MTC cell proliferation depends on PI3K/Akt/mTOR signaling, suggesting a novel therapeutic target for MTC [21]. A potent anti-proliferative effect of RAD001, mediated by the cell cycle arrest in G0/G1 phase, but not apoptosis, was recently shown in MTC cells [18]. To date, mTOR inhibitors failed to provide relevant benefits and patients often develop resistance to therapy and progression of disease [22]. Thus there are still unmet need for new therapeutic targets [23-25].

The chemokine CXCL12 binds the two receptors CXCR4 and CXCR7 and transduces on the mTOR pathway in pancreatic cancer, gastric cancer, T cell leukemia cell and in human renal cancer cells [26-28]. Chemokines are a superfamily of chemoattractant proteins that induce cytoskeletal rearrangement, firm adhesion to specific cells and directional migration [29]. The binding of chemokines to their receptors triggers a cascade of events, including receptor dimerization, recruitment of heterotrimeric G proteins, and activation of the Janus kinase and Signal Transducer and Activator of Transcription (STAT), PI3K and mitogen-activated protein kinases (MAPK), extracellular signal-regulated kinases (ERK) pathways. Emerging evidence indicates that chemokine receptors pathways control tumor development, including tumor growth, progression, and metastasis [30, 31] and that CXCL12/CXCR4 activation induces migration and metastatic processes [32-34].

The aim of the study was to evaluate the CXCR4/CXCL12/CXCR7 axis and the possible crosstalk with mTOR pathway in NETs to identify new prognostic and therapeutic targets.

## RESULTS

### CXCR4/CXCL12/CXCR7 pathway is overexpressed in GEP-NETs and MTCs

**Figure 1:**
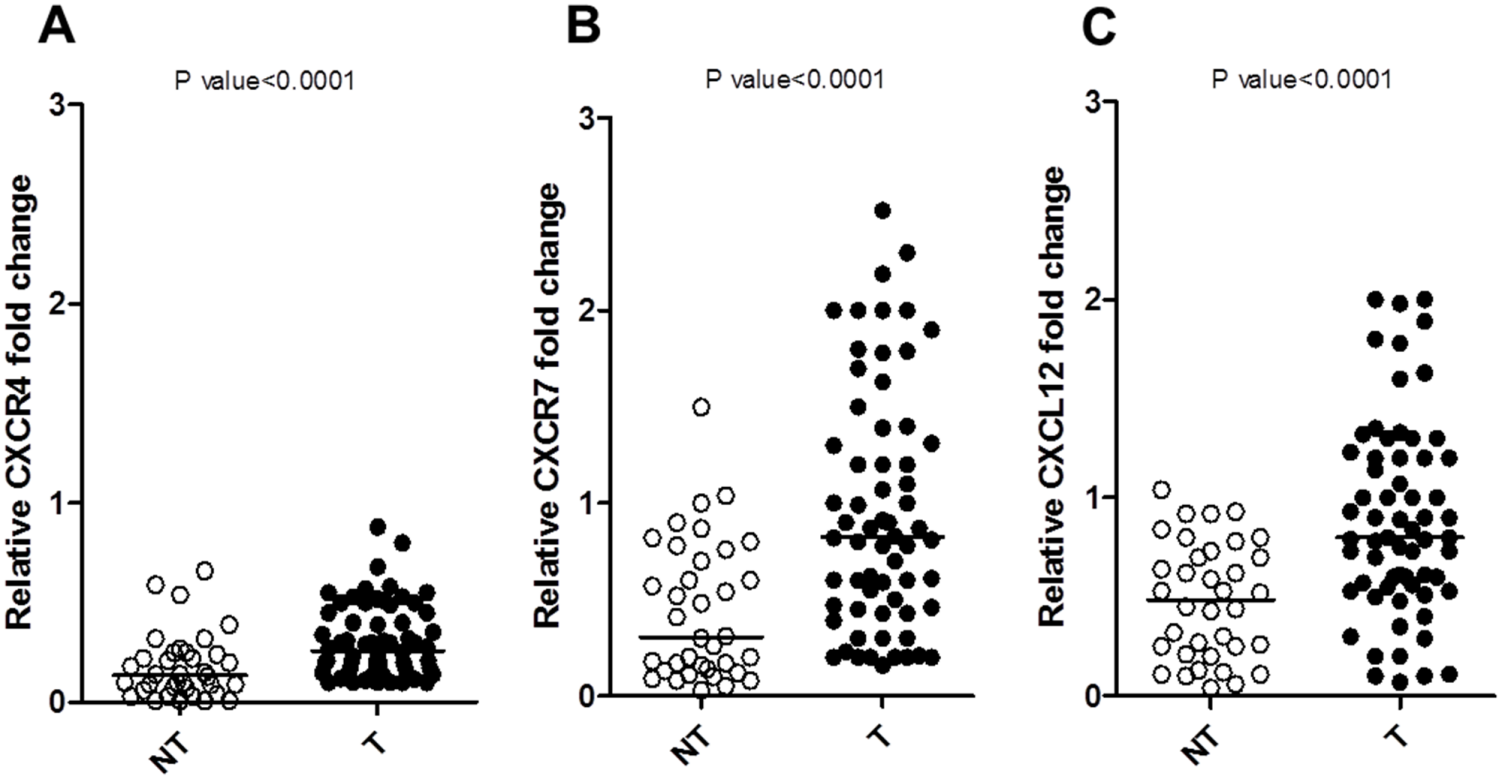
Relative mRNA expression of NETs (T) versus normal tissue (NT) by real-time RT-PCR **A.** CXCR4, **B.** CXCR7 and **C.** CXCL12 expression levels of T (Y axis) were calculated relative to the mean level of NT. All experiments have been performed in duplicate, and the average value of the results was plotted on the diagram. P value was calculated with Mann-Whitney test, two-tailed.

Clinical and pathological characteristics of the study population are summarized in Table 1. CXCR4, CXCL12 and CXCR7 mRNA expression was evaluated in 61 NETs and in 36 normal tissues, as shown in Figure 1. CXCR4, CXCL12 and CXCR7 were significantly overexpressed in NETs as compared to normal samples (p<0.0001).

**Table 1:** Clinicopathologic characteristics of patients with gastroenteropancreatic NETs and Medullary thyroid carcinoma

Characteristics	Numbers
***no. of patients***	61
***Median age in years (range)***	64.5 (28-86)
***Gender (Males/Females)***	27/34
***Primary tumor***	
Unknown	4
Thyroid	21
Colon	7
Appendix	2
Duodenum	2
Pancreas	14
Stomach	1
Ileum	10
***pTNM of MTC***	
Stage I (T1N0M0)	8
Stage II (T2-T3N0M0)	9
Stage III (T1-T3N1M0)	4
Stage IV	0
***Tumor grading of GEP-NETs and unknown***	
*G1*	11
*G2*	14
*G3*	15

**Figure 2:**
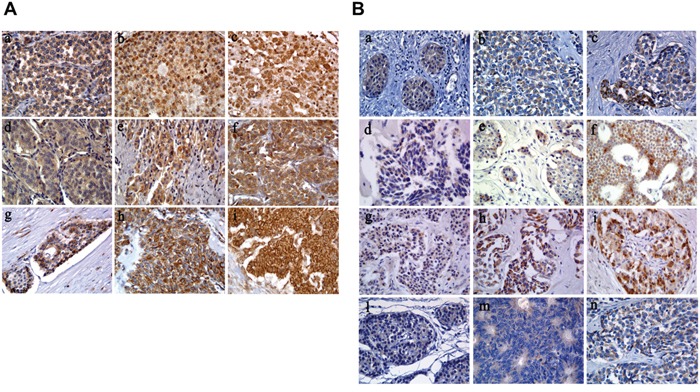
CXCR4/CXCL12/CXCR7 and mTOR pathway expressions in neuroendocrine tumor patients **A.** CXC immunostaining. NEC G3 stomach: weak CXCR4 (a), NET G2 ileum: moderate CXCR4 (b), NEC G3 colon: strong CXCR4 (c); NET G1 ileum: weak CXCR7 (d), NET G2 pancreas: moderate CXCR7 (e), NET G2 pancreas: strong CXCR7 (f); NET G1 ileum: weak CXCL12 (g), moderate CXCL12 (h), NEC G3 colon: strong CXCL12 (i) (original magnification, 400×). **B.** mTOR immunostaining. NET G1 appendix: weak mTOR (a), NEC G3 gastric: moderate mTOR (b), NEC G3 pancreas: strong mTOR (c); NET G1 pancreas: weak p-mTOR (d), NET G1 ileum: moderate p-mTOR (e), NET G1 ileum: strong p-mTOR (f); NETG2 ileum: weak p-S6K1 (g), NEC G3 colon: moderate p-S6K1 (h), NEC G3 pancreas: strong p-S6K1 (i); NET G1 appendix: weak p-4EBP1 (l), NET G2 colon: moderate p-4EBP1 (m), NEC G3 gastric: strong p-4EBP1 (n) (original magnification, 400×).

CXCR4, CXCL12 and CXCR7 protein levels were evaluated by IHC in 53/61 patients (31 GEP-NETs, 18 MTCs and 4 unknown). A positive immunostaining was found in 28/31 (90%) GEP-NETs and 13/18 (72%) MTCs for CXCR4, in 29/31 (93%) GEP-NETs and 17/18 (94%) MTCs for CXCR7 and in 30/31 (97%) GEP-NETs and 16/18 (89%) MTCs for CXCL12 (Table 2) (Figure 2).

**Table 2:** Immunostaining of CXC and mTOR factors in gastroenteropancreatic NETs and Medullary thyroid carcinoma

Protein	GEP-NETs	MTCs
	Positive %	Positive %
CXCR4	90	72
CXCR7	93	94
CXCL12	97	89
mTOR	58	56
p-mTOR	58	78
p-S6K1	61	67
p-4EBP1	52	50

A significant correlation was identified among CXCR4, CXCR7 and CXCL12 (Table 3). In GEP-NETs, a significant positive correlation was observed between the CXCR4/CXCL12/CXCR7 immunostaining score and Ki67 score (p<0.05). CXCR4 and CXCR7 were more expressed in G3 than in G1/G2 (p<0.05). In MTCs, CXCR7 and CXCL12 immunostaining was significantly higher in stage II-III as compared to stage I (p<0.05). Neither in GEP-NETs nor in MTCs, CXCR4/CXCL12/CXCR7 level correlated with clinical outcome.

**Table 3:** Spearman correlation of CXC and mTOR factors in gastroenteropancreatic NETs and Medullary thyroid carcinoma

	CXCR7	CXCL12	mTOR	p-mTOR	p-S6K1	p-4EBP1
CXCR4 *P*	**ρ = 0.415** **0.002**	**ρ = 0.298** **0.032**	**ρ = 0.395** **0.003**	ρ = 0.081 0.564	**ρ = 0.319** **0.020**	ρ = 0.103 0.481
CXCR7 *P*		**ρ = 0.418** **0.002**	ρ = 0.099 0.486	ρ = −0.053 0.711	**ρ = 0.285** **0.040**	ρ = 0.139 0.347
CXCL12 *P*			ρ = 0.248 0.077	ρ = −0.216 0.124	ρ = 0.070 0.624	ρ = 0.231 0.114
mTOR *P*				**ρ = 0.299** **0.029**	**ρ = 0.335** **0.014**	**ρ = 0.578** **<0.0001**
p-mTOR *P*					**ρ = 0.352** **0.010**	**ρ = 0.326** **0.022**
p-S6K1 *P*						**ρ = 0.312** **0.029**

The mTOR pathway was evaluated; 18 of 31 GEP-NETs (58%) were positive for both mTOR and p-mTOR, 10 (56%) and 14 (78%) of 18 MTCs were positive for mTOR and p-mTOR respectively. Moreover, 19/31 (61%) GEP-NETs and 12/18 (67%) MTCs were positive for p-S6K1, 16/31 (52%) GEP-NETs and 9/18 (50%) MTCs for p-4EBP1 (Table 2). As expected, a significant positive correlation was observed between mTOR and p-mTOR (p<0.05), mTOR and both p-S6K1 and p-4EBP1 (p<0.05 and p<0.0001) and between p-mTOR and its two effectors (p<0.05) (Table 3). The immunostaining score for p-mTOR (p<0.05) was significantly higher in G1/G2 tumors than in G3 in GEP-NETs, while it was not significantly correlated to tumor stage in MTCs. In the whole NET population, p-mTOR (p<0.01) and p-4EBP1 (p<0.01) scores were significantly high in patients with unfavorable outcome. In particular in GEP-NETs, p-mTOR (p<0.05), p-S6K1 (p<0.05) and p-4EBP1 (p<0.05) scores were high in patients with unfavorable outcome. In MTCs, there was no significant correlation between mTOR pathway immunostaining and outcome.

Finally, CXCR4 significantly correlated with mTOR (p<0.01) and p-S6K1 (p<0.05) while CXCR7 with p-S6K1 (p<0.05) (Table 3).

### CXCR4/CXCL12/CXCR7 axis signals on mTOR in NET cell lines

**Figure 3:**
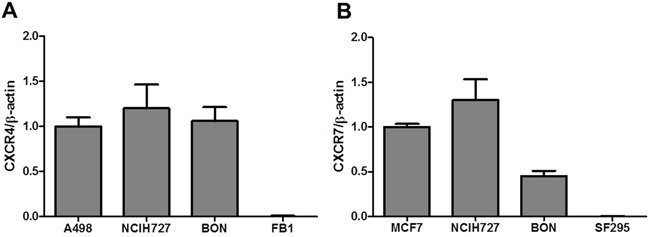
Relative mRNA expression in cell lines **A.** CXCR4 and **B.** CXCR7 mRNA expression was analyzed in NCI-H727 and BON through RT-PCR. A498 and FB1, human renal cancer and a human anaplastic thyroid cancer cell lines, as positive and negative control for CXCR4 respectively. MCF 7 and SF295, breast cancer cell line and a human glioblastoma, as positive and negative control for CXCR7 respectively.

**Figure 4:**
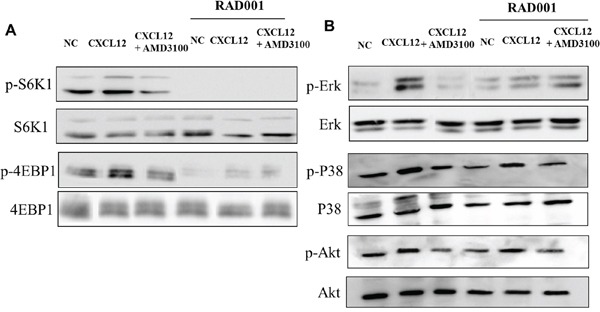
CXCR4 signals through the mTOR signaling pathway in BON cell line **A.** BON were stimulated with CXCL12 (100 ng/ml) for 10 min and after with AMD3100 (5 μM), with or without the RAD001 (1μM); p-S6K1 and p-4EBP1 protein expressions were measured using western blot analysis. Representative data from one of three experiments. **B.** p-Erk1/2, p-P38 and p-Akt activation was measured in the same conditions. Total Erk1/2, P38 and Akt proteins were used for normalization. Representative data from one of three experiments. NC: Normal control

To evaluate the CXCR4/CXCL12/CXCR7 axis in NET cell lines, NCI-H727 (bronchial-NET) and BON (pancreatic-NET) were tested for CXCL12 induced signaling. As shown in Figure 3, in BON and NCI-H727, CXCR4 and CXCR7 were overexpressed. CXCL12 induced the mTOR targets p-S6K1 and p-4EBP1 in BON cells and the CXCL12-induction was inhibited by the specific CXCR4 antagonist, AMD3100 (Figure 4). As expected, addition of mTOR inhibitor, RAD001, blocked the downstream mTOR effectors (Figure 4A); RAD001 modestly impaired CXCL12-mediated p-Erk1/2, p-P38 and p-Akt induction. Moreover CXCL12 induced the phosphorylation of Erk1/2 and P38, which was inhibited by AMD3100 (Figure 4B).

**Figure 5:**
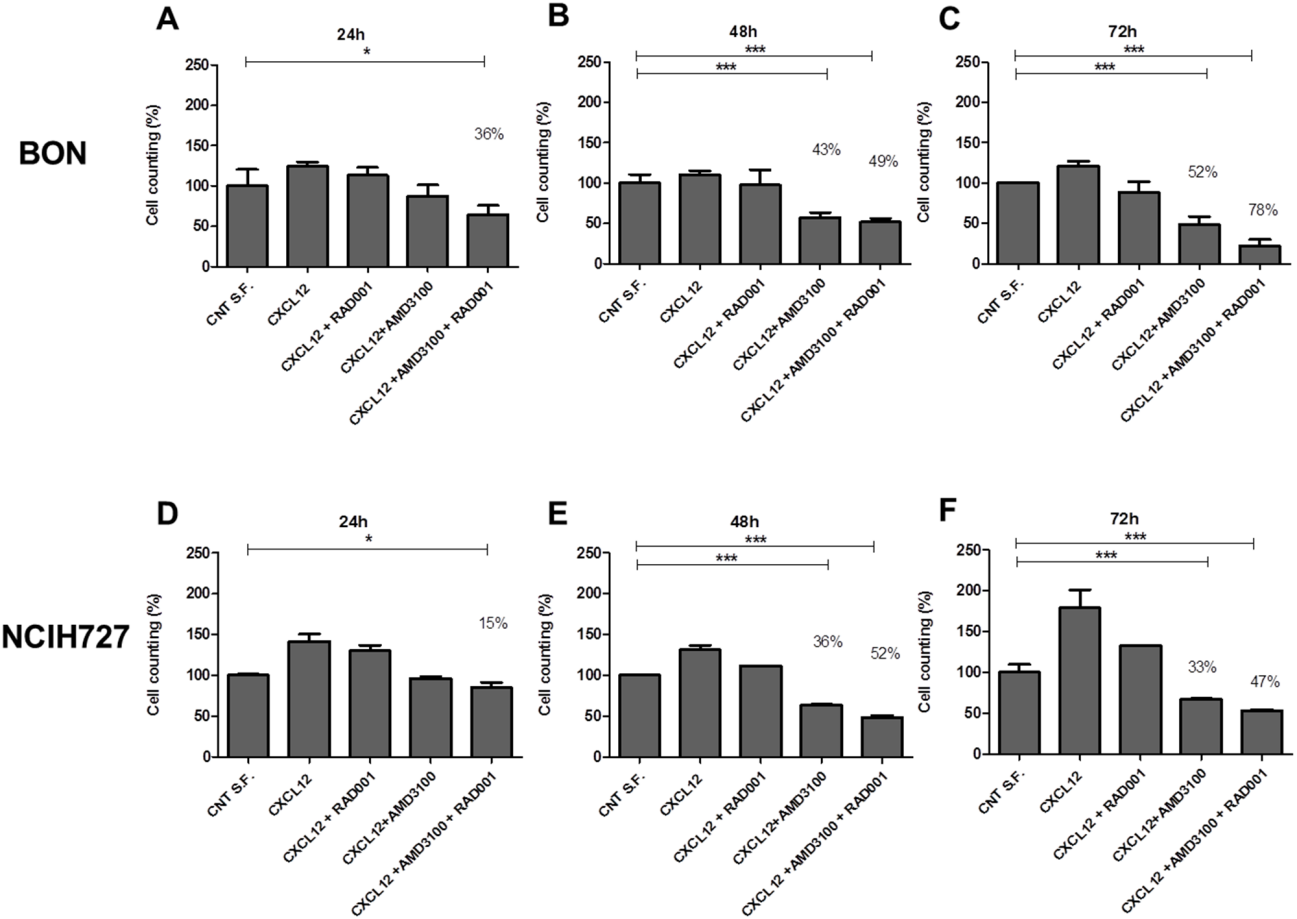
Effect of RAD001 and AMD3100 on cells proliferation BON after 24hrs **A.**, 48hrs **B.** and 72hrs **C.**; NCI-H727 after 24hrs **D.**, 48hrs **E.** and 72hrs **F.** in presence of CXCL12 (100 ng/ml) and AMD3100 (5 mM), with or without of RAD001 (1 μM). Results are representative of three different experiments performed. Each column represents the mean±S.D. Statistical significances were calculated by One-way ANOVA with Bonferroni correction. *P<0.5, **P<0.01, ***P<0.001. Control with serum free (CNT S.F.) versus treatments.

Then, the effects of CXCL12 were evaluated on the BON and NCI-H727 cells growth. AMD3100 and RAD001 reduced cell growth already at 24 hrs (Figure 5). Moreover, the addition of AMD3100 significantly (p<0.05) enhanced RAD001 anticancer activity after 24 hrs (Figure 5).

## DISCUSSION

To shed further insight on NET biology the role of the CXCR4/CXCL12/CXCR7 axis and the possible interconnection with the signaling on mTOR pathway was investigated. It was observed that CXCR4, CXCL12 and CXCR7 increased in both GEP-NETs and MTCs. Recent evidence demonstrated that CXCR4 is expressed in lung NETs [35] and significantly correlated with negative patient outcome [36]. To the best of our knowledge this is the first time that the whole CXCR4/CXCL12/CXCR7 axis was found overexpressed in NETs and correlating with tumor grading and Ki67 [37]. In MTCs, high CXCR7 and CXCL12 expression was detected in more advanced tumors and high p-mTOR level was observed in G1/G2 GEP-NETs compared to G3. The correlation between tumor grading and activation of p-mTOR in NETs is controversial [38, 39] probably because NETs are rare tumors but also because the poor differentiated NETs are underrepresented in the most of studies. Furthermore these data confirmed that the mTOR pathway is broadly expressed in NETs and seems prognostic factors [40]. Crosstalks between CXCR4, CXCL12 and PI3K/mTOR had been previously described in peritoneal disseminated gastric cancer [26], pancreatic cancer [41], human T-cel leukemia [42] and renal cancer [28]. The existence of an interplay between CXCR4, CXCL12 and PI3K/mTOR has been demonstrated in NETs, where CXCL12 induced p-S6K1 and p-4EBP1 in NET cell lines and the CXCL12-induction was inhibited by AMD3100. The addition of RAD001 blocked and further reduced the mTOR downstream effectors. In addition, at the immunohistochemical analysis, CXCR4 correlated with mTOR and p-S6K1 and CXCR7 with p-S6K1. As RAD001 impaired CXCL12-mediated p-Erk1/2 and p-P38 induction, the conclusion was that mTOR inhibition is downstream of CXCR4 axis.

CXCL12 dependent CXCR4 signals on mTOR inducing human NET cell growth while the inhibitors AMD3100 and RAD001 impaired cell growth with an additive effect. We recently developed a new class of cyclic peptidic CXCR4 antagonists [43], that could be evaluated in combination with RAD001in NET patients. The goal of this study will be to provide a dose and regimen for the combined treatment and its tolerability, as reported by other ongoing clinical trials exploiting the interaction of mTOR with other pathways [44].

In conclusion, to the best of our knowledge, this is the first report of CXCR4 expression and signaling in NETs through the mTOR pathway. CXCR4 may represent a new prognostic factor in NET tumors and mTOR inhibitors coupled with the CXCR4 inhibitors could represent a novel pharmacological approach to prevent mTOR drug resistance.

## MATERIALS AND METHODS

### Patients and specimens

Sixty-one patients diagnosed with NETs and followed in two Units of the Multidisciplinary NET group of Naples (the “Federico II” University Hospital and the Istituto Nazionale per lo Studio e la Cura dei Tumori, Fondazione “ G. Pascale” of Naples) were enrolled. Out of 61 patients, 53 tumor samples were available as paraffin embedded tissue (31 GEP-NETs, 4 unknown primary origin and 18 MTCs) and 8 as fresh tissue (5 GEP-NETs and 3 MTCs). GEP-NETs were classified according to the WHO International Histological Classification of Endocrine Tumors 2010 [37, 45] while MTCs were classified following the latest staging system (TNM) valid for thyroid tumors [7]. Normal tissue samples (7 thyroid, 29 gastrointestinal) were available for comparative studies.

Expression analysis by quantitative real-time PCR was performed on tumor samples of the whole NET population (53 paraffin embedded and 8 fresh tissues) and 36 normal tissue samples (28 paraffin embedded and 8 fresh tissues) while immunohistochemistry was performed on 53 tumors. Clinical course of disease after surgery was classified as favourable (no evidence of disease, no. 23) and unfavourable (disease persistence no. 11, death for tumor progression no. 10). Follow-up data were not available in 17 patients.

The study was approved by the institutional review board of the Institutions involved in the study (protocol n°102/2013 approved by the Federico II University Ethical Committee). Informed consent was obtained from patients for their tissues to be used in research.

The data were analyzed anonymously and all clinical investigation have been conducted according to the principles expressed in the Declaration of Helsinki.

### Immunohistochemistry

Immunohistochemistry (IHC) was performed using the primary monoclonal antibodies: CXCR4, CXCR7 and CXCL12 were from R&D Systems; mTOR, p-mTOR (Ser2448) and p-S6K1 (Thr389) were from Cell Signaling Technology; p-4EBP1 (Thr70) was from Abcam. The specificity of all reactions was validated in parallel with negative controls obtained omitting primary antibodies. Immunohistochemical findings were independently evaluated by four pathologists (EG, MDBDC, SL, FT). Cases with conflicting scores were reviewed jointly with a multi-head microscope until a consensus was reached. All cases were evaluated using a semi-quantitative scoring (IRS) system [46]. Staining intensity was scored as 0 (negative), 1 (weak), 2 (moderate), or 3 (strong) (Figure 1). The percentage of tumor cells stained were scored as 0 (none), 1 (1–10%), 2 (11–50%), 3 (51–80%), 4 (> 80%). The intensity and percentage were multiplied, resulting in an individual immunoreactivity score (IRS) ranging from 0 to 12. The raw expression scores were used for correlation analysis. For correlation with clinic pathological variables, any expression of CXCR4, CXCR7, CXCL12, mTOR, p-mTOR, p-S6K1 and p-4EBP1 (IRS 1–12) was considered positive.

Ki67 score was evaluated in GEP-NETs tumor samples by counting >2000 cells in immunohistochemical samples representative of the tumour, and a percentage of immunoreactivity (Labeling Index) was calculated [47]. The mitotic count was evaluated according to the method recommended in the 7th edition of the AJCC Cancer Staging Manual: enumerate mitoses in the most mitotically active area (“hot spot”) and then extend mitotic count to adjacent contiguous fields. If no mitotic activity was evident, random representative tumor fields were scanned for mitoses.

### Cell culture

Human NET cell lines used in this study include a Bronchial-NET cell line (NCI-H727) and a pancreatic-NET cell line (BON). NCI-H727 cell line was obtained from American Type Culture Collection (ATCC) and BON cell line was gifted by Professor Papotti (University of Turin at San Luigi Hospital, Orbassano).

NCI-H727 was maintained in Rosswell Park Memorial Institute (RPMI)-1640 and BON was cultured in Dulbecco's Modified Eagle Medium:Nutrient Mixture F-12 (DMEM/F12) both supplemented with 10% fetal bovine serum (FBS), 2 nM L-glutamine, penicillin 10, 000 IU/mL and streptomycin 10, 000 μg/mL (all from Sigma–Aldrich) in a humidified atmosphere containing 5% CO2 at 37°C. Human renal cancer cells (A498), human breast adenocarcinoma cells (MCF-7) and human glioblastoma cells (SF295) were obtained from the NCI-Drug Screen Program and human anaplastic thyroid cancer cells (FB-1) were obtained from the courtesy of Prof. Rosamarina Melillo, Federico II University, Naples-Italy.

### RNA extraction and quantitative real-time PCR

RNA was isolated from NETs fresh tissue or paraffin-embedded tissues using the RNeasy Mini kit (Qiagen, Hilden, Germany) and RNeasy FFPE Kit according to manufacturer's instructions. RNA was synthesized for cDNA using SuperScript III Reverse Transciptase (Invitrogen). Quantitative real-time PCR was performed using SYBR Green PCR Master Mix (Applied Biosystems, Foster City, CA). β-actin gene was used as an endogenous control for sample normalization. Data were collected and quantitatively analyzed on an ABI Prism 7000 System (Applied Biosystems).

The gene specific primers used for amplification were as follows:

CXCR4: Fw 5′-TGGGTGGTTGTGTTCCAGTTT-3′
Rw 5′-ATGCAATACCAGGACAGGATGA-3′,
CXCR7: Fw 5′-GATTGCCCGCCTCAGAAC-3′
Rw 5′-GCAGGACGCTTTTGTTGG-3′,
CXCL12: Fw 5′-TGTGGCACTCAGATACCGACT-3′
Rw 5′-CCCACAGAGCCAATCACT-3′.

A498 and MCF7 cell lines were used as CXCR4 and CXCR7 positive control, respectively; FB1 and SF295 as CXCR4 and CXCR7 negative control, respectively. Samples were run in triplicate, and their relative expression was calculated in the following formula using β-actin as endogenous control: 2^−ΔΔCt^.

### Western blot

BON cell line was allowed to grow in T75 flasks until 70% confluence. The CXCR4 inhibitor, AMD3100, was obtained from Sigma (St. Louis, MO, USA) and RAD001 from Novartis. Complete media was replaced with CXCL12 (100 ng/ml) for 10 min and after with AMD3100 (5 μM), with or without the RAD001 (1μM). After treatment, total protein was extracted from cells, after homogenization in lysis buffer (10 mM NaF, 10 mM Na-pyrophosphate, 1 mM Na3VO4. Invitrogen) containing protease and phosphatase inhibitors (Sigma-Aldrich). Then the supernatants were obtained using centrifugation at 4°C, 14 000 r.p.m. for 15 min. Total proteins (50 μg) were separated using Sodium Dodecyl Sulphate - PolyAcrylamide Gel Electrophoresis (SDS-PAGE) at 10%. Proteins on gels were transferred to Nitrocellulose Blotting Membranes 0.45 μm (GE Healthcare Life Sciences) for 1.5 hours at 220 mA in a transfer chamber. Membranes were blocked with 5% milk in TBST (10 mM Tris-HCl, pH 7.5, 200 mM NaCl, 0.05% Tween-20) for 1 hour at room temperature and then incubated overnight in primary antibodies at 4°C. Rabbit monoclonal antibodies for Erk1/2, p-Erk1/2 (Thr202/Tyr204), S6K1, p-S6K1 (Thr389), 4EBP1, p-4EBP1, p-P38 (Thr180/Tyr182), P38, p-Akt (Ser473), Akt were from Cell Signaling Technology. The non-phosphorylated form of each protein was used as internal control. Membranes were washed three times with TBST for 10 minutes and incubated with secondary antibody for 2 hours at room temperature. Secondary antibodies include goat anti-rabbit-HRP (Jackson ImmunoResearch). After 3 TBST washes of 10 minutes each, membranes were revealed through EMD Millipore Immobilon™ Western Chemiluminescent HRP Substrate (ECL).

### Proliferation assay

BON and NCI-H727 cells were plated into 6-well plates at a density of 50 × 10^4^ cells/well in duplicate. After 24 hrs, RAD001 (1μM), AMD3100 (5μM) or both agents were added. Cells were incubated for 24-48-72 hrs at 37°C in a humidified atmosphere containing 5% CO2 and counted using a hemocytometer.

### Statistical analyses

Statistical analysis was performed using Graphpad 5 software (Graphpad Software, La Jolla, CA, USA) and the results were considered statistically significant at a level of p<0.05. The t Tests, nonparametric Mann-Whitney test, one-tailed was used to analyze relative mRNA expression level of CXCR4/CXCL12/CXCR7 axis in tumors versus non-tumor tissues and it was used to compare the distribution of the markers investigated among the different tumor groups and with clinical and pathological variables. The Spearman's test was used to analyze the correlation index among markers expression. One-way ANOVA and multiple comparisons with Bonferroni correction was used to evaluate the growth cells in vitro.
